# Regulation of endothelial function by cigarette smoke and next**-**generation tobacco and nicotine products

**DOI:** 10.1007/s00424-023-02824-w

**Published:** 2023-06-07

**Authors:** Justus Klein, Patrick Diaba-Nuhoho, Sindy Giebe, Coy Brunssen, Henning Morawietz

**Affiliations:** 1 Department of Medicine III, Division of Vascular Endothelium and Microcirculation, Faculty of Medicine, University Hospital Carl Gustav Carus Dresden, TUD Dresden University of Technology, Fetscherstr. 74, D-01307 Dresden, Germany; 2 Department of Paediatric and Adolescent Medicine, Paediatric Haematology and Oncology, University Hospital Münster, Albert-Schweitzer-Str. 33, D-48149 Münster, Germany

**Keywords:** Cardiovascular diseases, Endothelial dysfunction, Cigarette smoking, Next-generation tobacco and nicotine products

## Abstract

Cigarette smoking is the most important avoidable cardiovascular risk factor. It causes endothelial dysfunction and atherosclerosis and increases the risk of its severe clinical complications like coronary artery disease, myocardial infarction, stroke, and peripheral artery disease. Several next**-**generation tobacco and nicotine products have been developed to decrease some of the deleterious effects of regular tobacco smoking. This review article summarizes recent findings about the impact of cigarette smoking and next**-**generation tobacco and nicotine products on endothelial dysfunction. Both cigarette smoking and next**-**generation tobacco products lead to impaired endothelial function. Molecular mechanisms of endothelial dysfunction like oxidative stress, reduced nitric oxide availability, inflammation, increased monocyte adhesion, and cytotoxic effects of cigarette smoke and next**-**generation tobacco and nicotine products are highlighted. The potential impact of short- and long-term exposure to next**-**generation tobacco and nicotine products on the development of endothelial dysfunction and its clinical implications for cardiovascular diseases are discussed.

## Introduction

Cardiovascular diseases are the major causes of death [102]. Tobacco smoking is the most important avoidable risk factor of cardiovascular diseases [103]. In 2019, more than 1 billion people were smokers consuming more than 7 trillion cigarette-equivalents of tobacco [30]. The prevalence of smoking has reduced by 27–38% in males and females since 1990, but due to the increase in global population, the total number of smokers has even further increased [30]. Therefore, smoking tobacco accounted for 7.7 million deaths and 200 million disability-adjusted life-years, and was the leading risk factor for death among males (20% of male deaths) in 2019 [30]. Cigarette smoking is a well-known risk factor of atherosclerosis and its life-threatening clinical complications like coronary artery disease, myocardial infarction, stroke, and peripheral artery disease [14, 29, 30, 80]. An important initial step in the development of atherosclerosis is endothelial dysfunction [62, 79].

In an attempt to decrease the deleterious effects of classical cigarette smoking, next**-**generation tobacco and nicotine products have been developed [69, 73, 92]. Next-generation tobacco and nicotine products include electronic (e) cigarettes and Heat-Not-Burn Tobacco products. Despite a partial reduction of deleterious components of classical cigarette smoke, also, these novel e-cigarettes, Heat-Not-Burn Tobacco products, and water pipe smoking promote endothelial dysfunction and cardiovascular diseases [69, 90, 104]. We would like to focus in this review first on the different components of cigarette smoke and next**-**generation tobacco products. Next, the impact of cigarette smoke and next**-**generation tobacco and nicotine products on endothelial dysfunction and cardiovascular diseases and its potential clinical implications will be discussed.

## Cigarette smoke and next-generation tobacco products

Cigarette smoke is an aerosol containing more than 4.700 components [37, 91] with reactive oxygen species (ROS) and carbon monoxide (CO) as important pathogenic constituents [77]. Further well-known substances found in cigarette smoke are nicotine, polycyclic aromatic hydrocarbons, and cadmium as well as other metals and substances like benzene, formaldehyde, or tar [17, 19, 21, 42, 48, 75]. Cigarette smoke is often subdivided into a particulate and vapor phase. The particulate phase is defined by the cigarette filters as they have an impact on components larger than one micrometer, whereas all components of the vapor phase are not being affected by filters [58]. Biologically and clinically relevant components of the gas phase include CO, acetaldehyde, formaldehyde, acrolein, nitric oxide (NO), and carbon dioxide. An estimate of 10^15^ radicals per puff could be detected within the gas phase. Due to the reaction of NO and ROS, both found in the gas phase, there is a possible increase of the radical count even after the average ROS lifetime of less than one second. These remarkable long-living radicals are still spin trapped from gas-phase smoke after more than 5 min [76]. Main components of the particulate phase are tar, defined as all particulate matter that is collected on a Cambridge filter pad other than water and nicotine, and nicotine itself [75]. Investigations using Cambridge filter pads showed a radical concentration of 10^17^/g within the particulate phase [77]. Another commonly used classification of cigarette emissions is the subdivision between mainstream and sidestream smoke. While mainstream smoke is directly inhaled during smoking, sidestream smoke originates from the glowing tip of conventional cigarettes with an up to 100-fold higher concentration of toxic substances. When investigating the health impact of second-hand smoking, sidestream smoke is of major relevance as 85 % of passively inhaled smoke consists of sidestream cigarette smoke [53, 81].

The first designs of alternative tobacco products were developed in the 1960s. The urge to develop potentially less harmful tobacco products further increased in the 1980s with the evaluation of the Cancer Prevention Study I & II (CPS-I & CPS-II) showing a direct link between smoking and carcinogenesis [74, 98]. Even while these new tobacco products were not launched to the market, e.g. British American Tobacco (BAT) developed a heating tobacco product already in 1962 [22, 82]. Since 2007, electronic tobacco products (like e-cigarettes) have been available on the market [45]. Today, a variety of different electronic noncombustible tobacco products are commercially available. They are summarized using the term “next**-**generation tobacco products” (NGP’s) [43]. Among the large numbers of noncombustible NGP’s, two main groups can be divided: e-cigarettes and Heat-Not-Burn Tobacco products (HnB-TP’s). E-cigarettes, often referred as vaporizers, can be refilled with nicotine-containing liquids in different flavors. In HnB-TP’s, common tobacco is electronically heated to 250-350 °C, rather than burned at temperatures of up to 900 °C in conventional cigarettes [9, 74].

In 2016, the first HnB-TP, referred to as Tobacco Heating System 2.2 (THS 2.2), was available for purchase. As conventional combustive cigarettes, HnB-TP’s such as IQOS® (produced by Philip Morris International Inc.), use tobacco sticks with a tobacco part and a filter through which smoke is inhaled. The tobacco part is made of 70 % tobacco, water, glycine as humectant and aerosol promoting ingredient, flavorings and binders. Using these ingredients, a thin tobacco sheet is produced and rolled for better heating properties. The filter piece consists of a polymer portion needed to cool the aerosol and a cellulose acetate mouthpiece to imitate conventional cigarettes [52, 74]. The heating process is regulated by the holder and terminates automatically, for example in the IQOS® device after 6 min or 14 puffs, to prevent pyrolysis.

The smoking habit of any tobacco product is mainly dependent on the nicotine delivery as nicotine is the addictive substance [45]. Analysis of mainstream smoke of reference cigarettes and the HnB-TP IQOS® showed no difference between these products [7]. Furthermore, comparison of the nicotine blood concentrations have shown no significant differences in nicotine peak concentration and metabolization between IQOS® and conventional cigarettes [10]. Nicotine reduction was not the main aim for the development of NGP’s, but the reduction of toxic and carcinogenic substances. Different studies showed a reduction of tar by 35-50 % (depending on the experimental settings) in mainstream smoke of HnB-TP’s and a 2-fold reduction of CO as the main component of the vapor phase [7, 61]. The mainstream smoke concentration of highly carcinogenic tobacco-specific nitrosamines (such as N-nitrosonornicotine, N′-nitro-soanatabine, N-nitrosoanabasin, and nicotine-derived nitrosamine-ketone) was reduced 8- to 22-fold compared to conventional cigarettes [7, 59, 61, 85]. Furthermore, the concentration of carbonyl compounds (such as formaldehyde, acetaldehyde, acrolein, and crotonaldehyde) is reduced by 80–97 % in HnB-TP’s [74]. The formation of ROS as mediators of oxidative stress was also reduced in the vapor and the particulate phase of HnB-TP’s. However, despite an 80 % reduction in H_2_O_2_ mainstream smoke concentration, the consumption of one pack IQOS tobacco sticks per day increases the ROS intake by more than 4 times compared to inhaling urban air (reference cities were New York, USA & Seoul, Korea) [83].

## Impact of cigarette smoke and next-generation tobacco and nicotine products on endothelial dysfunction and cardiovascular diseases

**Fig. 1:**
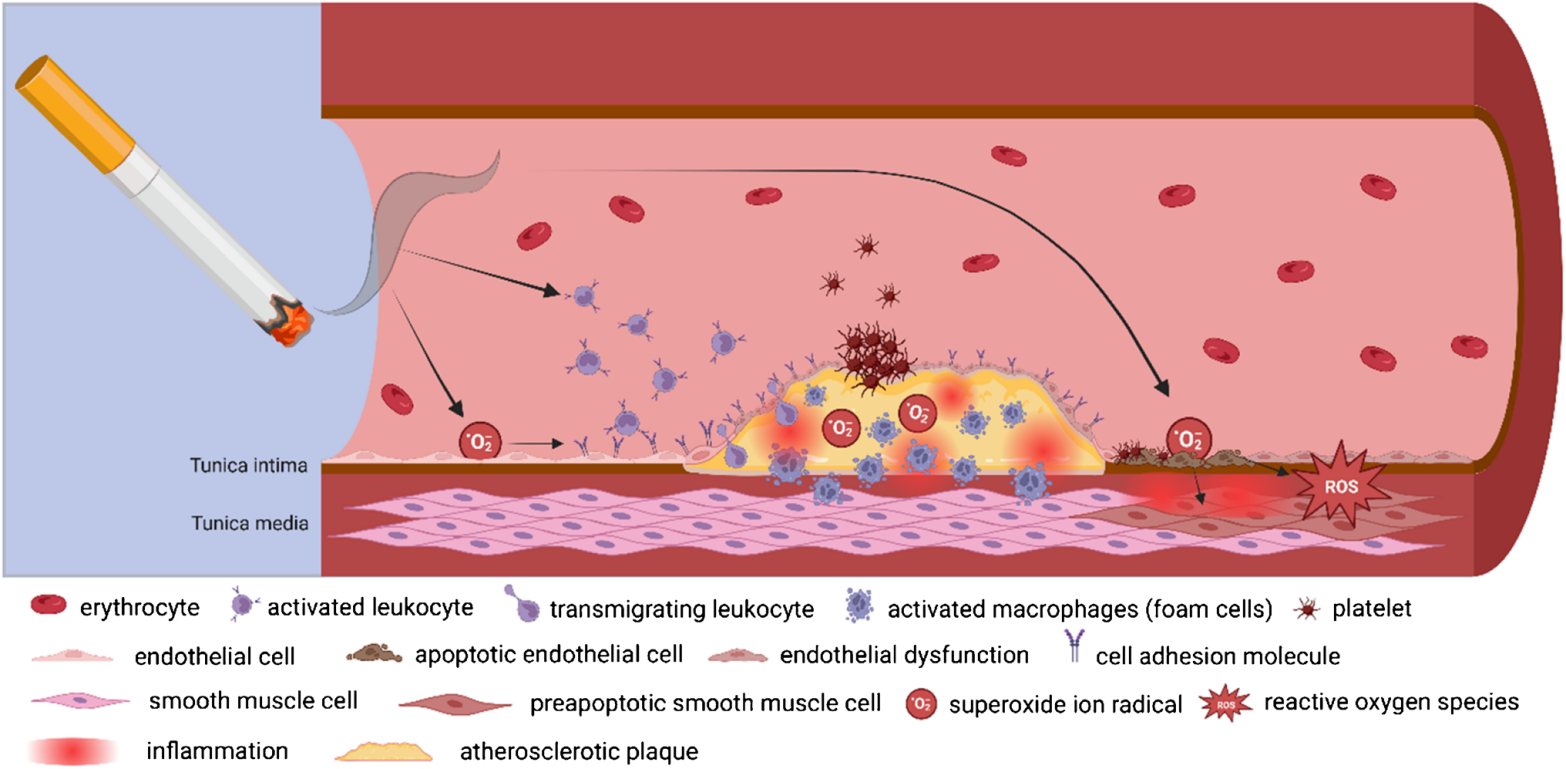
Impact of smoking on endothelial function. Smoking affects the vasculature in different ways. An increased level of superoxide ions could induce endothelial dysfunction leading to endothelial apoptosis. Cigarette smoke components are known to activate leukocytes and to induce the expression of cellular adhesion molecules in endothelial cells. This supports the adhesion and transmigration of leucocytes into the subendothelial space thus promoting atherosclerotic plaque formation and rupture leading to platelet-induced thrombus formation. Figure created with BioRender.com

Molecular mechanisms of endothelial dysfunction involve oxidative stress, reduced NO availability, inflammation, increased monocyte adhesion [4, 24, 39, 68], and cytotoxic effects of cigarette smoke and next-generation tobacco and nicotine products (Fig. 1). Although nicotine is mainly responsible for the addiction to cigarette smoking, the oxidative smoke fraction is responsible for the oxidative stress-induced development of endothelial dysfunction and atherosclerosis [17, 18, 34, 77]. However, the in vivo concentration of ROS is not only elevated by inhaled and pulmonary absorbed components but also due to several mechanisms leading to increased endogenous ROS production. Major sources of ROS like nicotinamide adenine dinucleotide phosphate (NADPH) oxidases or xanthine oxidase can be induced by cigarette smoke [51]. α,β-unsaturated ketones and a number of saturated aldehydes as well as α,β-unsaturated aldehydes, such as acrolein and crotonaldehyde, can be found in cigarette smoke and are known to induce NADPH oxidase isoforms [44] leading to increased generation and release of superoxide anions. ROS can also be found in cigarette smoke and react with NO released by endothelial nitric oxide synthase 3 (eNOS) in endothelial cells forming peroxynitrite. The oxidation of (6R)-5,6,7,8-tetrahydro-L-biopterin, a cofactor of the eNOS [27, 31], leads to uncoupling of the eNOS enzymes regularly acting as dimers [26, 57]. When functioning as a monomer, superoxide anion is being released as an electron is transferred to O_2_ during oxidation, therefore increasing the ROS concentration within the endothelium [35]. Circulating blood cells are affected by an increased ROS concentration due to smoking even before endothelial cells. Leukocytes release ROS, when mice are exposed to cigarette smoke [95]. Monocytes are known to express cellular adhesion molecules, when they are activated by cigarette smoke [49]. A transmigration of activated leukocytes (such as monocytes) is furthermore supported by an increased endothelial expression of intercellular adhesion molecule 1 (ICAM1), endothelial leukocyte adhesion molecule 1 (E-selectin), and vascular cell adhesion molecule 1 (VCAM1) due to cigarette smoke components interacting with the endothelium and a cigarette smoke-dependent activation of the pro-inflammatory transcription factor nuclear factor kappa-light-chain-enhancer of activated B-cells (NF-κB) [12, 88]. These changes shift the endothelial phenotype from the physiological anti-thrombotic state to a pro-thrombotic and pro-inflammatory state [11]. Thrombus formation is further supported by the increased number of thrombocytes in smokers [23]. In addition, next-generation tobacco products can promote thrombosis [3]. Ultimately, smoking can alter the stability of preexisting atherosclerotic plaques by activating matrix metalloproteinases and increasing the risk of plaque rupture [17, 72] leading to acute coronary syndrome [66].

In previous own studies, we analyzed the impact of cigarette smoke extract and next-generation tobacco and nicotine products on parameters of endothelial function [33, 34]. Cigarette smoking extract from reference cigarettes reduced endothelial cell viability in a dose-dependent manner. On the molecular level, cigarette smoking extract activated the nuclear factor erythroid 2-related factor 2 (NRF2) and its target genes heme oxygenase (decycling) 1 (HMOX1) or NAD(P)H quinone dehydrogenase 1 (NQO1) [33]. This supports a transient cellular adaption to cigarette smoke-induced oxidative stress. The atheroprotective activation of the Akt/eNOS pathway and improved wound healing in response to high laminar flow in human endothelial cells were inhibited by cigarette smoke extract [33]. Furthermore, cigarette smoke extract induced pro-inflammatory endothelial adhesion molecules and the adhesion of monocytes to endothelial cells under pro-atherosclerotic low flow conditions [33]. In a follow-up study, we analyzed parameters of endothelial function in response to aqueous smoke extracts of a heated tobacco product (HTP), an electronic cigarette (e-cig), a conventional reference cigarette (3R4F), and nicotine under different flow conditions [34]. All nicotine products activated anti-oxidative or pro-inflammatory responses in endothelial cells [34]. Next-generation nicotine product effects were typically lower compared to classical cigarette smoke extract. Furthermore, cigarette smoke extract impaired endothelial wound healing and induced a pro-inflammatory phenotype in comparison to next-generation tobacco and nicotine products [34]. More recently, we could show that cigarette smoking extract, aqueous smoke extracts of a heated tobacco product, an electronic cigarette, a conventional cigarette (3R4F), and pure nicotine activated anti-oxidative and pro-inflammatory processes in human monocytes [32]. Next-generation tobacco and nicotine products mediated lower responses relative to controls than monocytes exposed to cigarette smoke extract [32]. These in vitro data suggest a slightly reduced potential of next-generation tobacco and nicotine products to induce endothelial dysfunction in comparison to classical cigarette smoking. The activation of NRF2 and the upregulation of cytochrome p450 in response to cigarette smoke extract, but not to electronic cigarette aerosol extract in human coronary endothelial cells supports this concept [96]. Recently, a novel Nrf2-OSGIN1&2-HSP70 axis has been described that regulates endothelial adhesion and elevates GDF15 and HSP70 as novel biomarkers of plaque erosion in patients who smoke [84]. These recent studies shed new light into the molecular mechanisms of endothelial dysfunction in response to cigarette smoke extract and next-generation tobacco and nicotine products.

Several other experimental and clinical studies analyzed the impact of cigarette smoke extract and next-generation tobacco and nicotine products on endothelial function. Cigarette smoke extract mediates cytotoxic effects on human endothelial cells by reducing cell viability and inducing markers of apoptosis like cleaved caspase-3 and necrosis [63]. Cigarette smoke extract and its major cytotoxic component acrolein increased oxidative stress and reduced endothelial nitric oxide expression and activity [17, 40]. Heat-not-burn cigarette smoke extract decreased mitochondrial metabolic activity in human vascular endothelial cells [41]. Endothelial nitric oxide synthase activity was reduced by nicotine- and tar-free cigarette smoke extract of commercial devices like IQOS and hi-lite, but not Ploom S and glo [41]. Flavoured tobacco products are a major reason for the increasing popularity of next-generation tobacco products. However, even low concentrations of selected flavours (e.g. vanillin, menthol, cinnamaldehyde, eugenol, and acetylpyridine) can induce inflammation and impair the endothelial nitric oxide as markers of endothelial dysfunction [25]. The impact of selected components of cigarette smoke and next-generation tobacco/nicotine products on endothelial function is shown in Table 1. Potential effects of cigarette smoke and next-generation tobacco/nicotine products on the vascular wall are summarized in Table 2.

**Table 1:** Impact of selected components of cigarette smoke and next-generation tobacco/nicotine products on endothelial function (modified after [28, 74])

Substance	Unit	HnB-tobacco sticks [64]	Conventional cigarettes [16]	Reduction (%)	Impact on endothelial function
Nicotine	mg/tobacco sample*	1.1	1.07–2.70	-	Promotes endothelial dysfunction and release of catecholamines [20] and causes hemodynamic changes (e.g. alteration of heart rate and blood pressure, vasoconstriction) [5, 8, 36]
Acetaldehyde	μg/tobacco sample	179.4–183.5	930–1540	80.7–88.5	Inhalation of acetaldehyde gases at smoke-relevant concentrations impairs flow-mediated dilation (FMD) by 50 % [71]
Acrolein	μg/tobacco sample	8.9–9.9	89.2–154.1	90.0–94.2	Promotes endothelial dysfunction, oxidative stress, dyslipidemia, and platelet activation [13, 20, 55, 89, 93, 100]
Formaldehyde	μg/tobacco sample	4.7–5.3	29.3–130.3	84.0–96.4	Induces endothelial dysfunction [47]
Crotonaldehyde	μg/tobacco sample	<3.0	32.7–70.8	90.8–95.8	Induces vascular injury via DNA interstrand crosslinks, glutathione perturbation, mitogen–activated protein kinase, and Wnt and ErbB signaling pathways [105] and at higher concentrations tension oscillations (spasms) and irreversibly impaired contractility [46]
Benzene	μg/tobacco sample	0.5–0.6	49.7–98.3	99.0–99.5	Increases low-density lipoprotein, decreases circulating angiogenic cells, and increases cardiovascular risk scores [1]
1,3 Butadiene	μg/tobacco sample	0.2	77.0–116.7	99.7–99.8	Promotes oxidative stress and atherosclerosis [78]

*Tobacco samples are defined as one tobacco stick of HnB tobacco products and one conventional cigarette. The studies used the Health Canada Intense (HCI) protocol

**Table 2:** Potential effects of cigarette smoke and next-generation tobacco/nicotine products on the vascular wall

Effects on vascular wall	Cigarette smoke	Next-generation tobacco/nicotine products
Oxidative stress	Induces oxidative stress	Induces lower level of oxidative stress
Reduced NO availability	Reduces NO availability	Less impact on NO availability
Inflammation	Activates and promotes inflammation	Activates and induces lower levels of inflammation
Increased monocyte adhesion	Increases monocyte adhesion to endothelium	Reduced impact on monocyte adhesion
Cytotoxic effects	Causes cytotoxic effects on endothelial cells	Cytotoxic effects may be milder
Endothelial phenotype	Shift of endothelial phenotype to a pro-thrombotic and pro-inflammatory state	Could affect endothelial phenotype
Thrombus formation	Supports thrombus formation	Could support thrombus formation
Plaque stability	Alters stability of preexisting atherosclerotic plaques, increased risk of plaque rupture	Could affect plaque stability

## Clinical implications

The impact of next-generation tobacco and nicotine products on endothelial function in clinical studies is still not well-understood. First studies analyzed the effects of short-term exposure to e-cigarettes. Vaping e-cigarettes did not induce changes in heart rate, systolic and diastolic blood pressure, endothelial function (measured by flow-mediated dilation), and arterial stiffness (determined by cardio-ankle vascular index) in a 2-h clinical study in young, healthy, tobacco product naïve participants [15]. However, in animal models aerosol from a single “heat-not-burn” product (IQOS) exposure impaired endothelial function (flow-mediated dilation) to the same extend as by cigarette smoke [70]. This is supported by a recent meta-analysis indicating that acute inhalation of e-cigarettes leads to impaired endothelial function [65]. In populations who cannot give up smoking, “heat-not-burn” products can reduce biomarkers of vascular inflammation, oxidative stress, and endothelial dysfunction [6]. Recent studies suggest that no single constituent of smoke is responsible for the acute impairment of endothelial function [71]. Instead, acute endothelial dysfunction by inhaled products is caused by vagus nerve signaling initiated by airway irritation [71]. In addition, chronic vaping and smoking lead to impaired flow-mediated dilation by inhibition of endothelial NO release [67]. Finally, vaping increases microvascular endothelial permeability and affects the balance of pro- and anti-oxidative processes [67]. RAGE could be in this context a novel mediator of e-cigarette-mediated endothelial dysfunction [67].

New tobacco products were developed as less harmful alternatives to cigarette smoking. They were also considered potential supporting strategy in smoking cessation. Quitting smoking will remain the most effective way to reduce the negative health impact of cigarette smoke and to improve endothelial function. Long-term studies have shown that the cardiovascular risk (including prevalence of coronary heart disease, heart failure, and mortality) of former smokers is equal to the cardiovascular risk of never-smokers after >15 years of smoking cessation [2, 97]. Current therapies of nicotine addiction include behavioral or/and medical treatment [87]. Studies investigating the effectiveness of behavioral interventions identified giving brief advice (duration < 1min) [94], counseling (in groups or individually) [56], contingency management [38], or text messaging [38, 101] as possible methods to treat patients. A first-line medical therapy, known to support acute withdrawal, treat cravings as well as to reduce the relapse risk, is a combination of varenicline with different short-acting nicotine patches [99]. In this context, the concept of using new tobacco products to support smoking cessation is still controversially discussed [86]. While the prevalence of classical cigarette smoking has been slightly reduced in the last decades, the use of next-generation tobacco products has increased to a similar degree. In the recent Population Assessment of Tobacco and Health Study, increasing rates of discontinuing cigarette smoking and smokeless tobacco use were accompanied by decreasing rates of discontinuing electronic nicotine delivery systems use among youth in the USA [50].

A recent meta-analysis suggest among patients who attempt to quit smoking, e-cigarettes might be more efficacious than conventional nicotine replacement or behavioral smoking cessation therapies [60]. However, there is currently little evidence supporting effective vaping cessation interventions and no evidence for dual use cessation interventions [54].

In summary, several experimental and clinical data suggest specific deleterious effects of cigarette smoke and next-generation tobacco and nicotine products on endothelial function. Both cigarette smoking and next-generation tobacco products lead to impaired endothelial function. Additional experimental and clinical studies will lead to a better understanding of the underlying molecular mechanisms of the regulation of endothelial function in response to single, short-term, and long-term exposure to cigarette smoke and next-generation tobacco and nicotine products.

## Data Availability

Not applicable.
